# Tracheostomy Wound Myiasis in a Child: Case Report and Review of the Literature

**DOI:** 10.1155/2012/317862

**Published:** 2012-01-11

**Authors:** J. Blejter

**Affiliations:** Pediatric Surgery Service, Sanatorio Trinidad de Quilmes, Provincia de Buenos Aires, B1878CBI Quilmes, Argentina

## Abstract

An 8-year-old tracheostomized, gastrostomized, and with cerebral palsy boy was admitted for tracheostomy wound myiasis and cellulitis. Ether was applied in the wound, and then all the accessible larvae were removed. Antibiotic therapy was initiated. The procedure had to be repeated three more times to extract all the larvae. An airway endoscopy was performed and ruled out the presence of larvae in the airway, as well as any damage to the wall of the trachea. The patient recovered uneventfully and was discharged.

## 1. Introduction

Myiasis is a very common disease, especially in the developing world. But here, in a very unusual location, myiasis is presented.

## 2. Methods

The methods used were chart review of the patient and research of the related literature using the following keywords: “tracheostomy,” “myiasis,” and “treatment.”

## 3. Results

**Figure 1:**
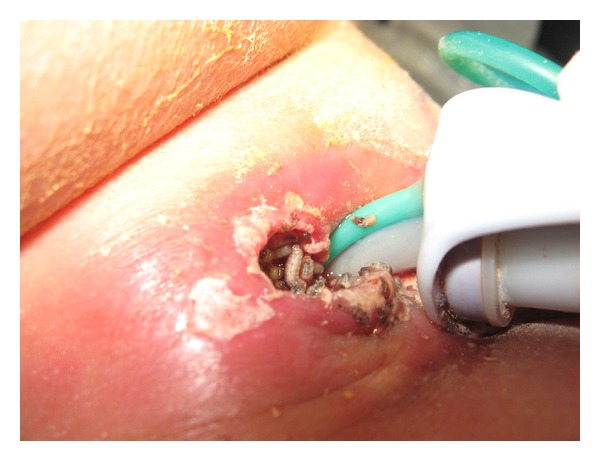
Tracheostomy wound myiasis.

An 8-year-old tracheostomized, gastrostomized, and with cerebral palsy boy who was admitted for tracheostomy wound myiasis in the right lateral aspect of it and cellulitis (Figure 1) is presented.

There were multiple larvae, which were 5 mm long and 1 mm wide. Antibiotic therapy was started and ether was applied to the wound, and then all the accessible larvae were removed. The procedure had to be repeated three more times to extract all the larvae (which at the last procedure measured 10 mm by 3 mm). In the first procedure, the cannula was replaced for a new one, and a larva was found at the balloon area, so an airway endoscopy was performed and ruled out the presence of larvae in the airway, as well as any damage to the wall of the trachea. Probably the larva was pulled out from the wound as the cannula was exiting.

The patient recovered uneventfully and was discharged. At the time of publication, the wound was healing properly.

## 4. Discussion

Myiasis is a common disease in Argentina and other developing countries [1–3]—especially tropical ones. It can affect any place of the anatomy, with the skin being the most affected one. Tracheostomy wound myiasis is very unusual, and there are very few case-report articles published about it [2, 4, 5]. 

Myiasis can be classified as furunculoid, subcutaneous infestation with tunnel formation, subcutaneous infestation with migratory swellings, and wound infestation [3].

Furunculoid myiasis is generally caused by *D. hominis *and* C. anthropophaga*. It consists of a single or multiple cutaneous nodules that contain just one larva, they are painful and pruriginous most of the times, while fever and secondary infections may also occur.

Subcutaneous infestation with tunnel formation presents itself with painful and pruritic serpiginous lesions. It must be differentiated from cutaneous larva migrans, but lesions in the latter are thinner, erythematous, and more pruritic than myiasis and rapidly moving; in addition, they are generally located in the lower extremity.

Subcutaneous infestation with migratory swellings presents similarly to both the subcutaneous infestation with tunnel formation and the furunculoid kind, because they produce the tunnel, but usually ends up in furunculoid lesions from which the larva emerge.

Clinical manifestations of wound infestation myiasis are visible larvae in the wound and secondary infection. Sometimes lesions get bigger due to destruction of vital and necrotic tissue. Flies responsible for wound myiasis are *C. hominivorax, C. bezziana, L. sericata *and* M. domestica*.

Morbidity is related to the place of the myiasis. Secondary infection is one of the most common complications, and they are resolved with antibiotics and larvae removal. In the case of nasal, otic, and ophthalmomyiasis death rate can be as high as 5% due to affection of the central nervous system. Ophthalmomyiasis can lead to pseudoorbital cellulitis and blindness in the affected eye [2, 6].

There are predisposing factors, [2–4] such as poor hygiene and poor socioeconomic environment, but this patient did not suffer any of these. In fact, most indexed articles are from developed countries. Although this does not reflect the real incidence in the developing world, we must be alerted that it can happen in spite of ideal conditions.

The treatment of wound infestation myiasis is usually quite simple. One should apply a substance that deprive the larvae from oxygen (ether, Vaseline, etc.), so they have to emerge to the surface and then they can be removed [1–4, 7]. However, in the furunculoid and almost in every migratory myiasis, surgical removal may be needed because the larva does not always emerge, remaining in the subcutaneous tissue and producing inflammation, infection, and granulomas [2, 3]. That is also why care should taken to remove the entire larva. Antiparasitic drugs, such as ivermectin, have also been used as treatment for this kind of myiasis [4, 8].

Prevention of this disease is accomplished by proper dressing of wounds, good hygiene, and preventing flies from entering houses, hospitals, and so forth. Ironing destroys eggs that may be left on the cloth by female flies. Also, treating infected animals can help prevent myiasis [2–4].

In conclusion, myiasis is generally an easy-to-treat-and-diagnose disease, although rarely there can by high morbidity and even deadly cases. Prevention, especially in developing countries, is hard.
